# Differential Effects of D-Galactose Supplementation on Golgi Glycosylation Defects in TMEM165 Deficiency

**DOI:** 10.3389/fcell.2022.903953

**Published:** 2022-05-26

**Authors:** Zoé Durin, Marine Houdou, Willy Morelle, Lydia Barré, Aurore Layotte, Dominique Legrand, Mohamed Ouzzine, François Foulquier

**Affiliations:** ^1^ University of Lille, CNRS, UMR 8576—UGSF—Unité de Glycobiologie Structurale et Fonctionnelle, Lille, France; ^2^ Faculty of Medicine, UMR7365 CNRS-University of Lorraine, Biopôle, Nancy, France

**Keywords:** CDG, TMEM165-CDG, glycosylation, galactose, manganese

## Abstract

Glycosylation is a ubiquitous and universal cellular process in all domains of life. In eukaryotes, many glycosylation pathways occur simultaneously onto proteins and lipids for generating a complex diversity of glycan structures. In humans, severe genetic diseases called Congenital Disorders of Glycosylation (CDG), resulting from glycosylation defects, demonstrate the functional relevance of these processes. No real cure exists so far, but oral administration of specific monosaccharides to bypass the metabolic defects has been used in few CDG, then constituting the simplest and safest treatments. Oral D-Galactose (Gal) therapy was seen as a promising tailored treatment for specific CDG and peculiarly for TMEM165-CDG patients. TMEM165 deficiency not only affects the *N-*glycosylation process but all the other Golgi-related glycosylation types, then contributing to the singularity of this defect. Our previous results established a link between TMEM165 deficiency and altered Golgi manganese (Mn^2+^) homeostasis. Besides the fascinating power of MnCl_2_ supplementation to rescue *N*-glycosylation in TMEM165-deficient cells, D-Gal supplementation has also been shown to be promising in suppressing the observed *N*-glycosylation defects. Its effect on the other Golgi glycosylation types, most especially *O*-glycosylation and glycosaminoglycan (GAG) synthesis, was however unknown. In the present study, we demonstrate the differential impact of D-Gal or MnCl_2_ supplementation effects on the Golgi glycosylation defects caused by TMEM165 deficiency. Whereas MnCl_2_ supplementation unambiguously fully rescues the *N*- and *O*-linked as well as GAG glycosylations in TMEM165-deficient cells, D-Gal supplementation only rescues the *N*-linked glycosylation, without any effects on the other Golgi-related glycosylation types. According to these results, we would recommend the use of MnCl_2_ for TMEM165-CDG therapy.

## Introduction

Glycosylation is a universal and fundamental cellular process in all living organisms leading to a broad variety and diversity of glycan structures on proteins and lipids, all having specific physiological functions. The biosynthesis of these structures is tightly regulated and defects in any of the glycosylation pathways lead to severe metabolic diseases named Congenital Disorders of Glycosylation (CDG) (Jaeken and Carchon, 1993; Ondruskova et al., 2021). Identified in 2012, TMEM165-CDG present a very specific and unique clinical phenotype of bone defects classified as spondylo-epi-(meta)-physeal dysplasia (Zeevaert et al., 2012). More precisely, TMEM165-CDG patients exhibit a short stature associated with dwarfism, scoliosis, and severe osteoporosis with very thin bone cortex and dysplastic vertebrae, ribs and toenails. Defects in TMEM165 have been associated with strong Golgi glycosylation abnormalities affecting different glycosylation types (*N*-; *O*-; glycolipids and glycosaminoglycans (GAGs)), mainly characterized by hypo-galactosylated glycan structures and largely contributing to the unique clinical phenotype of TMEM165-deficient patients (Potelle et al., 2016). Since Golgi β-1,4-galactosyltransferase depends on Mn^2+^ for its activity (Ramakrishnan et al., 2004) we postulated that the hypo-galactosylation of glycans observed in TMEM165-CDG patients arises from impaired Mn^2+^ transport by TMEM165 into the Golgi (Potelle et al., 2016). Mn^2+^ supplementation would thus permit to restore adequate Mn^2+^ concentration in the Golgi for Golgi β-1,4-galactosyltransferase activity, as well as for that of the vast majority of other Golgi glycosyltransferases, also dependent on Mn^2+^ (Lairson et al., 2008).

The molecular mechanism linking glycosylation defects to the loss of TMEM165 emerged from the keystone result that MnCl_2_ supplementation in TMEM165-CDG patients cells completely suppresses the observed Golgi glycosylation defects (Potelle et al., 2016; Houdou et al., 2019). Such treatment is now envisaged for TMEM165-CDG patients as MnCl_2_/MnSO_4_-H_2_O supplementation was shown to be successful for two patients suffering from another CDG, SLC39A8-CDG (Park et al., 2018). SLC39A8 was reported as one the major Mn^2+^ transporters of the plasma membrane of cells whose defect in SLC39A8-CDG patients leads to very low to undetectable Mn^2+^ concentrations in blood and causes intracellular Mn^2+^ deficiency (Choi et al., 2018); reviewed in Foulquier and Legrand (2020). As such, TMEM165-CDG together with SLC39A8-CDG are the two first CDGs in which Golgi glycosylation defects result from a primary disorder in Mn^2+^ homeostasis. Beneficial effects of Mn^2+^ administration led to major clinical improvements and corrected all biochemical abnormalities in the SLC39A8-CDG patients. Interestingly, the link between TMEM165 and Mn^2+^ homeostasis was further emphasized by our observation that TMEM165 is specifically degraded in lysosomes in response to high extracellular Mn^2+^ concentration (Potelle et al., 2017). The cellular functions of TMEM165 are not completely deciphered yet, but there is now a substantial body of evidence that supports TMEM165 as a novel Golgi Ca^2+^-Mn^2+^/H^+^ exchanger regulating both Golgi Ca^2+^ and Mn^2+^ homeostasis.

Besides Mn^2+^ supplementation, our work highlighted that D-galactose (Gal) supplementation in TMEM165-CDG could also normalize the observed *N*-glycosylation defects (Morelle et al., 2017), albeit partially compared to Mn^2+^. Interestingly, it was also shown that D-Gal supplementation is able to normalize glycosylation defects found on serum transferrin from SLC39A8-CDG patients (Park et al., 2015). The mechanism by which exogenous D-Gal addition suppresses some of the Golgi glycosylation defects is unknown but D-Gal therapies have also been proven to be successful in the treatment of PGM1-CDG and SLC35A2-CDG (Morava, 2014; Witters et al., 2017; Witters et al., 2020). Oral administration of monosaccharides are widely used in CDG and are seen as the simplest and safest treatments. For instance, L-fucose has been used for SLC35C1-CDG and D-mannose for MPI-CDG (Brasil et al., 2018; Sosicka et al., 2020; Verheijen et al., 2020).Nonetheless, whereas D-Gal supplementation partially rescues the *N*-glycosylation in TMEM165 deficiency (Morelle et al., 2017), its effect on the other glycosylation types, especially *O*-GalNAc glycosylation and synthesis of GAGs, is still unknown. It has thus been investigated in the present study. Our results actually highlight that D-Gal supplementation has a rather poor effect on the Golgi glycosylation defects observed in TMEM165 deficiency, as sole the *N*-glycosylation is rescued. In contrast, MnCl_2_ supplementation fully overcomes all the different Golgi glycosylation defects. Knowledge of the uniqueness of D-Gal supplementation for the *N*-glycosylation is crucial for setting up a TMEM165-CDG therapy.

## Material and Methods

### Cell Culture

Control, TMEM165 KO [generated by Pr Lupashin’s lab and described in (Vicogne et al., 2020)] and COSMC KO (generated by Pr Wandall’s lab) HEK293 cells were maintained in Dulbecco’s Modified Eagle’s Medium (DMEM) (Lonza, Basel, Switzerland) supplemented with 10% fetal bovine serum (FBS) (PAN, Germany), at 37°C with 5% CO_2_ and humidity-saturated atmosphere. ATDC5 cells (Riken cell, Tsukubai, Japan) were cultured in DMEM-F12 complete medium (2 mM glutamine, 100 μg/ml streptomycin, 100 IU/ml penicillin) supplemented with 5% FBS. For drug treatments, HEK cells were incubated either with MnCl_2_ from Riedel-de-Haën (Seelze, Germany) and/or D-Galactose (Sigma-Aldrich, St Louis, MO, United States), 50 nM thapsigargin, or 100 µM cyclopiazonic acid as described in each figure and their legends. ATDC5 cells were cultured in DMEM F12 complete medium until reaching 80% confluency. Then, the medium was replaced with DMEM-F12 without FBS and containing 1 μM of MnCl_2_, 1 mM of D-Galactose or 1 mM of xylose for 36 h.

### Plasmids and Transfection

Decorin cDNA was generated by PCR and cloned into EcoRI and BamHI or SmaI and PstI sites of pCMV empty vector (Stratagene, Valencia, CA). For transfection, cells were seeded in 6-well plates until 80% confluency and transfected with 1 µg of either pCMV-Decorin, or pCMV-empty vector using lipofectamine 2000 transfection reagent (Invitrogen, Carlsbad, CA) according to manufacturer’s instructions. Expression of decorin in culture medium was analyzed 48 h post-transfection by western blotting using anti-decorin specific antibodies.

### Lectin Staining

Cells were seeded on glass coverslips in 6-well plates and incubated in the above-described cell culture conditions for 24 h. Cells were then washed three times with Dulbecco’s Phosphate Buffer Saline containing magnesium and calcium (DPBS^+/+^) (Sigma Aldrich) and fixed with 4% paraformaldehyde (PAF) for 20 min at room temperature. PAF was neutralized by 50 mM NH_4_Cl [Sigma–Aldrich (St Louis, MO, United States)] for 10 min at room temperature. Neuraminidase from *Vibrio Cholerae* (Sigma Aldrich) was diluted at 1/60 in PBS (Euromedex) and incubated for 1 h on coverslips, at 37°C. VVL-fluorescein and PNA-Cy5 lectins (Vector Laboratories) were diluted at 2 μg/ml in PBS containing 0.1% bovine serum albumin (BSA) (Roche Diagnostics, Penzberg, Germany) and added on coverslips for 1 h in a humid atmosphere in the dark. Coverslips were then washed three times with PBS and stained with 5 μg/ml DAPI in PBS for 10 min. After one wash with PBS, coverslips were rinsed in deionized water before being mounted on glass slides with Mowiol. Fluorescence was detected through an inverted Zeiss LSM780 confocal microscope. Acquisitions were performed using the ZEN pro 2.1 software (Zeiss, Oberkochen, Germany).

### Western-Blot

Cells were scrapped in PBS (Euromedex) and centrifugated at 4000 g at 4°C for 10 min. Cell pellets were lysed with RIPA buffer (Tris/HCl 50 mM pH 7.9, NaCl 120 mM, NP40 0.5%, EDTA 1 mM, Na_3_VO_4_ 1 mM, NaF 5 mM) supplemented with a protease cocktail inhibitor (Roche Diagnostics, Penzberg, Germany), and centrifugated at 20,000 g, 4°C for 30 min. Protein concentration in the supernatant was estimated with the micro–BCA Protein Assay Kit (Thermo Scientific). 10 µg of protein were mixed with NuPAGE LDS sample buffer (Invitrogen), pH 8.4, supplemented with 4% β-mercaptoethanol (Fluka). Samples were denaturated for 10 min at 95°C, separated on 4%–12% Bis-Tris gels (Invitrogen) and transferred onto nitrocellulose membranes using a iBlot2 Dry Blotting System (Thermo Fisher Scientific, Waltham, MA United States) for 7 min, according to manufacturers’ indications. Membranes were blocked in blocking buffer (5% milk powder in 1X TBS-T (Euromedex) and 0.05% Tween20 (Euromedex)) for 1 h at room temperature, then incubated overnight at 4°C with the primary antibodies in blocking buffer and washed three times for 5 min in TBS-T. Membranes were then incubated with peroxidase-conjugated secondary goat anti-rabbit or goat anti-mouse antibodies (Dako; used at a dilution of 1:10,000 or 1:20,000) or donkey anti-sheep antibodies in blocking buffer for 1 h at room temperature and later washed three times for 5 min in TBS-T. Signal was detected with chemiluminescence reagent (ECL 2 Western Blotting Substrate or SuperSignal West Pico PLUS chemiluminescent Substrate, Thermo Scientific) on imaging film (GE Healthcare, Little Chalfont, United Kingdom) or Camera Fusion® (Vilber Lourmat) and its software.

Mouse anti–β-actin antibody was purchased from MilliporeSigma (Burlington, MA, United States) and used at 1:10,000 dilution. Mouse anti-LAMP2 antibody from Santa Cruz Biotechnology (Dallas, TX, United States) was diluted at 1:2000 and sheep anti-TGN46 (Biorad) at 1:1000. Decorin antidoby from R&D Systems (Mineapolis, MN, United States) was diluted at 1:1000.

### Deglycosylation Assay

Cells were lysed in RIPA buffer supplemented with sodium fluorure and orthovanadate, and proteins were quantified as previously described using a micro–BCA Protein Assay Kit (Thermo Scientific). The deglycosylation assay was performed with the Agilent Enzymatic Deglycosylation Kit for *N*-Linked and Simple *O*-Linked Glycans (GK80110) and Agilent Extender Kit for Complex *O*-Linked Glycans (GK80115), according to manufacturer’s instructions (Agilent, Santa Clara, CA 95051 United States). In brief, 100 µg of protein (in a maximum volume of 30 µL) were heated with 10 µL of incubation buffer and 2.5 µL of denaturation buffer at 100°C for 5 min. After the cooling down, 2.5 µL of detergent solution and 1 µL of one or more deglycosylation enzyme (depending on the condition wanted) were added to the mix. Samples were incubated at 37°C for 3 h. The adequate volume for 10 µg of protein of each sample was used for further western-blotting analysis.

### Benzyl-GalNAc

Cells were seeded in T75 flasks. When they reached 80% confluency, the medium was changed to DMEM supplemented with 5% FBS, 250 µM benzyl-GalNAc (Bn-GalNAC) (GalNAcαBnSigma) and, if needed, MnCl_2_ and D-galactose. Cells were incubated for 3 days before collecting medium.

Medium was run on 10 kDa high-mass cut-off [Amicon, from MilliporeSigma (Burlington, MA, United States)] for 30 min at 2,460 g. Flow-through was freeze-dried, and the powder was resuspended in ATFA (Sigma Aldrich) 0.1%, and run over Sep-pak C18 cartridge (S*Pure Pte Ltd., Singapore). Columns were equilibrated first with methanol, then, ATFA 0.1%, acetonitrile and again ATFA 0.1%. Resuspended flow-through was applied and then the column was washed with 0.1% ATFA. Bn-GalNAc glycans were eluted with a 1:1 ratio of acetonitrile and ATFA 0.1%. Samples were dried under an azote flux, and freeze-dried.

Dried samples were permethylated by addition of 400 µL of DMSO [Sigma–Aldrich (St Louis, MO, United States)] and 200 mg of dry NaOH [Sigma–Aldrich (St Louis, MO, United States)] and shaken for 30 min at 4065 g. 1 ml of milliQ water and 1 ml of chloroform were added to stop the reaction. Samples were vortexed and the aqueous phase was removed. Five more washes with 1 ml of water were performed before evaporating chloroform under an azote flux. Permethylated samples were resuspended in 30 µL of a 1:1 ratio of water and methanol. 1 µL of resuspended sample was mixed with 1 µL of DHB matrix (10 mg/ml-Sigma-Aldrich, MO, United States), before being and spotted on a plate and analyzed on Shimadzu Biotech Axima Resonnance MALDI QUIT-TOF.

### Statistical Analysis

Comparisons between groups were performed using Student t-test for two variables with equal or different variances, depending on the result of the F-test. *t*-Test have been made with GraphPad, using raw data of three independent experiments. *p* values are indicated in legends, as well as the number of experimental replicates.

## Results

### Differential Impact of D-Galactose Treatment on the Suppression of LAMP2 and TGN46 Glycosylation Defects

**FIGURE 1:**
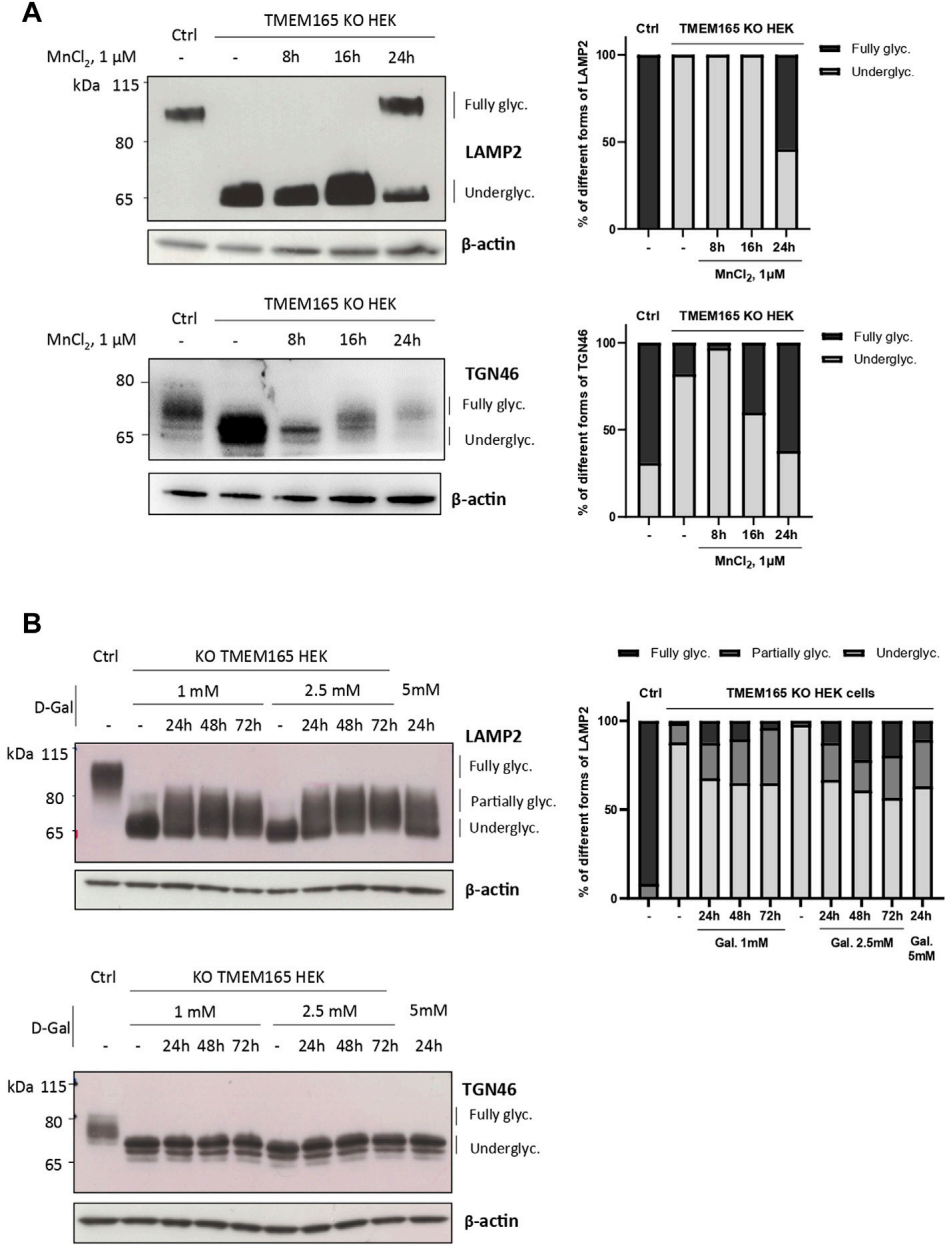
Differential impact of Mn^2+^ and D-Galactose treatments on LAMP2 and TGN46 glycosylation profiles in TMEM165 KO HEK cells. **(A)**. Western-blots of LAMP2 and TGN46 in control (Ctrl) and TMEM165 KO HEK cells, treated with 1 µM MnCl_2_ for 8, 16 and 24 h. Graphs show the relative quantification of fully and underglycosylated forms of LAMP2 and TGN46. **(B)**. Western-blots of LAMP2 and TGN46 in control (Ctrl) and TMEM165 KO HEK cells, treated with 1 or 2.5 mM D-galactose (D-Gal) for the indicated times. In both panels, the electrophoretic migration positions of the fully- and under-glycosylated forms of proteins are indicated on the right side. The graph shows the relative quantification of fully, partially and underglycosylated forms of LAMP2 (*n* = 3).

**FIGURE 2:**
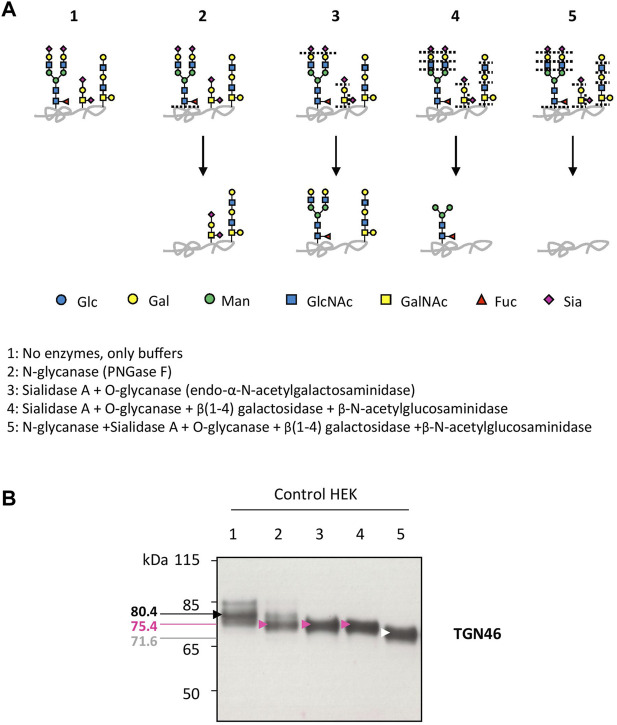
Insights into the nature of TGN46 glycosylation sites. **(A).** Schematic representation of the *N*- and *O*-linked glycan structures resulting from the enzymatic deglycosylation conditions described in the legend to scheme (1–5). Specific cutting sites are indicated with dashed lines. **(B).** Control HEK cell lysates were prepared for the different enzymatic digestions following guidelines and protocol provided by the manufacturer (see Material and Methods section). Then, samples were loaded onto SDS-PAGE and western-blot was incubated with TGN46 antibody. Black, pink, white and red arrows indicate different shifts in the migration of the major band within TGN46 electrophoretic pattern. Corresponding molecular weights are indicated on the left side of the western-blot. Gal, galactose; GalNAc, *N*-acetylgalactosamine; Glc, glucose; GlcNAc, *N*-acetylglucosamine; Fuc, fucose; Man, mannose and Sia, sialic acid (*n* = 3)

Our previous studies abundantly reported that LAMP2 glycosylation defects found in TMEM165 KO HEK cells could be suppressed by the addition of either MnCl_2_ or D-Gal in the culture medium (Morelle et al., 2017; Potelle et al., 2017; Houdou et al., 2019). To determine the specificity of such suppression, the migration profile of TGN46, another glycoprotein carrying *N*- and *O-*linked glycans was followed. While MnCl_2_ supplementation slowed down the electrophoretic gel mobilities of both TGN46 and LAMP2 in TMEM165 KO HEK cells (Figure 1A), D-Gal supplementation had no effect on TGN46 migration profile when compared to the presence of partially glycosylated forms of LAMP2 (Figure 1B). One can note that only a subset of LAMP2 glycosylation was rescued due to the slow turnover of LAMP2 estimated to 48 h in our previous study (Houdou et al., 2019). This was observed whatever the concentration (from 1 to 5 mM) or the incubation time with D-Gal (from 24 to 72 h) (Figure 1B). This result led us to investigate the nature of the glycosylation carried by TGN46. To do so, a set of different glycosidases such as *N*-glycanase (PNGase F), *O*-glycanase (endo-α-N-acetylgalactosaminidase), β(1,4)galactosidase, β-N-acetylglucosaminidase and sialidase A were used in HEK control cells (Figure 2A) and TGN46 migration profile was analyzed. As shown in Figure 2B, PNGase treatment, which removes *N*-glycan structures, led to a slight shift in the electrophoretic migration of TGN46 somehow revealing the presence of *N*-glycans on the protein (lane 2). The combined action of *O*-glycanase and sialidase A (lane 3), which removes core 1 *O*-glycan structures, resulted in a major shift in TGN46 migration profile, similar to the one observed after PNGase treatment (lane 2). The result in lane 4 indicates the absence of substituted and elongated *O-*glycans. At last, the action of both PNGase and *O*-glycanase (lane 5) led to a more pronounced shift in TGN46 electrophoretic migration. Altogether, these different treatments demonstrate that in addition of being *N*-glycosylated, TGN46 is also *O*-glycosylated.

### Strong *O*-Glycosylation Defects in TMEM165 KO HEK Cells Are Only Partially Rescued Following D-Gal Supplementation

**FIGURE 3:**
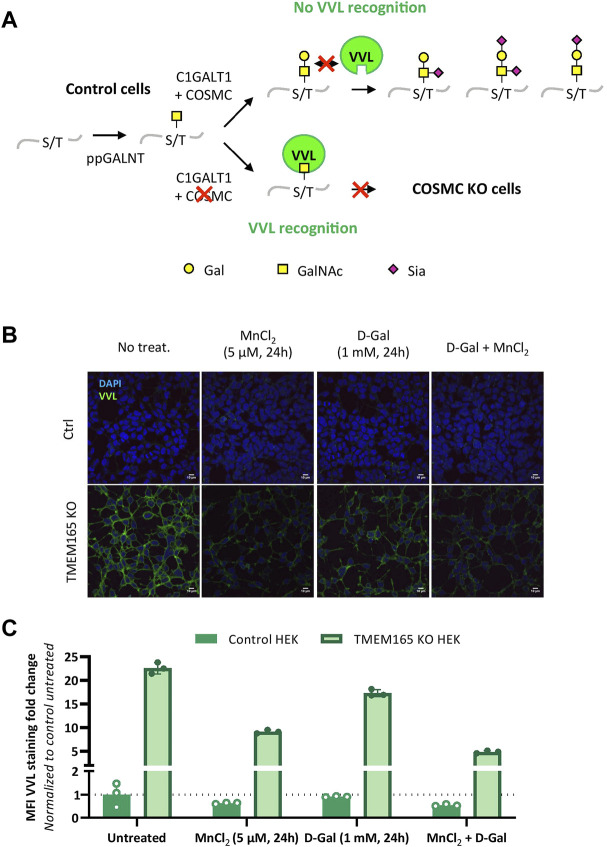
*O*-linked mucin-type defects in TMEM165 KO HEK cells. **(A).** Schematic representation of the initiating steps of core 1 mucin-type *O*-glycan synthesis and specific lectin recognition. First, a *N*-acetylgalactosamine (GalNAc) residue is linked to the hydroxyl group of a serine (S) or threonine (T) within a polypeptide *via* the activity of several peptidyl GalNAc transferases (ppGALNT). Then, the galactosyltransferase C1GALT1 catalyzes the addition of a galactose (Gal) residue onto GalNAc for further elongation by acid sialic (Sia). C1GALT1 requires the chaperone COSMC to be fully functional. VVL only recognizes free terminal GalNAc residues. In case of COSMC deficiency, GalNAc residues are no longer substituted and thus accessible to VVL. Symbols used to represent the different sugar residues are indicated at the bottom of the scheme. **(B).** Influence of MnCl_2_ and/or D-Gal supplementations on VVL-fluorescein staining in TMEM165 KO HEK cells. Cells were treated with the indicated treatments, fixed and labeled with VVL-fluorescein (green) before confocal microscopy visualization. DAPI staining (blue) was performed, showing nuclei. **(C).** Relative quantification of VVL-fluorescein staining in cells. The results are expressed as mean fluorescence intensity (MFI) percentages obtained after normalization of the VVL-fluorescence intensity to that of DAPI and considering the MFI of untreated control HEK cells as 1 (*n* = 3; *p* value < 0.001).

The lack of D-Gal effect on TGN46 migration profile, as well as the *O*-glycosylated nature of TGN46, led us to investigate the impact of TMEM165 deficiency on *O*-linked glycosylation. A lectin staining strategy using *Vicia villosa* lectin (VVL) coupled to fluorescein was used to study the *O*-glycosylation in TMEM165 KO HEK cells. VVL preferentially recognizes α-terminal GalNAc residues linked with *O*-glycosidic bonds to serine or threonine in polypeptides (Bard and Chia, 2016). This first GalNAc residue (also called Tn antigen) initiates the mucin-type *O*-linked glycans and is usually substituted by Gal and/or additional monosaccharides, in the Golgi apparatus (Figure 3A). As a positive control, VVL-fluorescein staining was first performed in COSMC KO HEK cells, COSMC being the C1GALT1-specific chaperone required for the proper activity of core 1 synthase galactosyltransferase 1 (C1GALT1), that catalyzes the transfer of Gal from UDP-Gal onto the first *O*-linked GalNAc residue (illustrated in Figure 3A). As shown in Supplementary Figure S1A, the green signal associated to VVL-fluorescein was significantly higher in COSMC KO HEK cells than in control cells, consistent with the presence of higher amounts of free terminal *O*-GalNAc residues expressed at the plasma membrane of COSMC KO HEK cells (Ju and Cummings, 2002). Interestingly, a similar VVL staining was observed in TMEM165 KO HEK cells (Supplementary Figure S1A), demonstrating the significant presence of truncated *O*-linked mucin type glycans in TMEM165-deficient cells. To then assess the effects of MnCl_2_ and/or D-Gal supplementations, control and TMEM165 KO HEK cells were treated with 2.5 μM MnCl_2_ and/or 1 mM D-Gal for 24 h. As depicted in Figures 3B,C, a significant decrease of the mean fluorescence intensity (MFI) associated to VVL-fluorescein was observed in TMEM165 KO cells treated in any of the three conditions. However, it is to note that D-Gal supplementation is much less efficient (17.3-fold change) compared to MnCl_2_ supplementation alone (9.1 fold) or to MnCl_2_ in combination with D-Gal (4.9 fold). This result not only demonstrates the presence of truncated mucin-type glycans in TMEM165 KO HEK cells, but also confirms that D-Gal supplementation has a limited rescuing effect on *O*-glycosylation deficiency compared to MnCl_2_ supplementation. This was also confirmed by PNA lectin staining that recognizes unsialylated terminal galactose residues on *O*-glycans. As expected, the PNA staining after D-Gal supplementation and de-sialylation (neuraminidase treatment) was fainter in TMEM165 KO cells than in control cells, then supporting impaired galactosylation in TMEM165-deficient cells (Supplementary Figure S1B). These results confirm the VVL staining result and demonstrate that D-Gal supplementation alone has a poor effect in rescuing the transfer of Gal onto *O*-GalNAc residues in TMEM165 KO HEK cells.

To confirm these results at the structural level, we took advantage of benzyl-α-GalNAc (Bn-GalNAc), an artificial substrate of Golgi-resident glycosyltransferases that mimics the precursor GalNAcα1-*O*-Ser or Thr in *O*-glycoproteins (Kudelka et al., 2016). Cells incubated with Bn-GalNAc will incorporate it within the Golgi. The resulting Benzyl-α-*O*-glycans processed by Golgi glycosyltransferases will be then further secreted and represent the cellular *O*-glycan capacities of the cell. To further assess the impact of MnCl_2_/D-Gal treatment on *O*-glycosylation, Bn-GalNAc was added to the culture media of control and TMEM165 KO HEK cells, cultured in different conditions, and its glycosylation status was analyzed by mass spectrometry.

**FIGURE 4:**
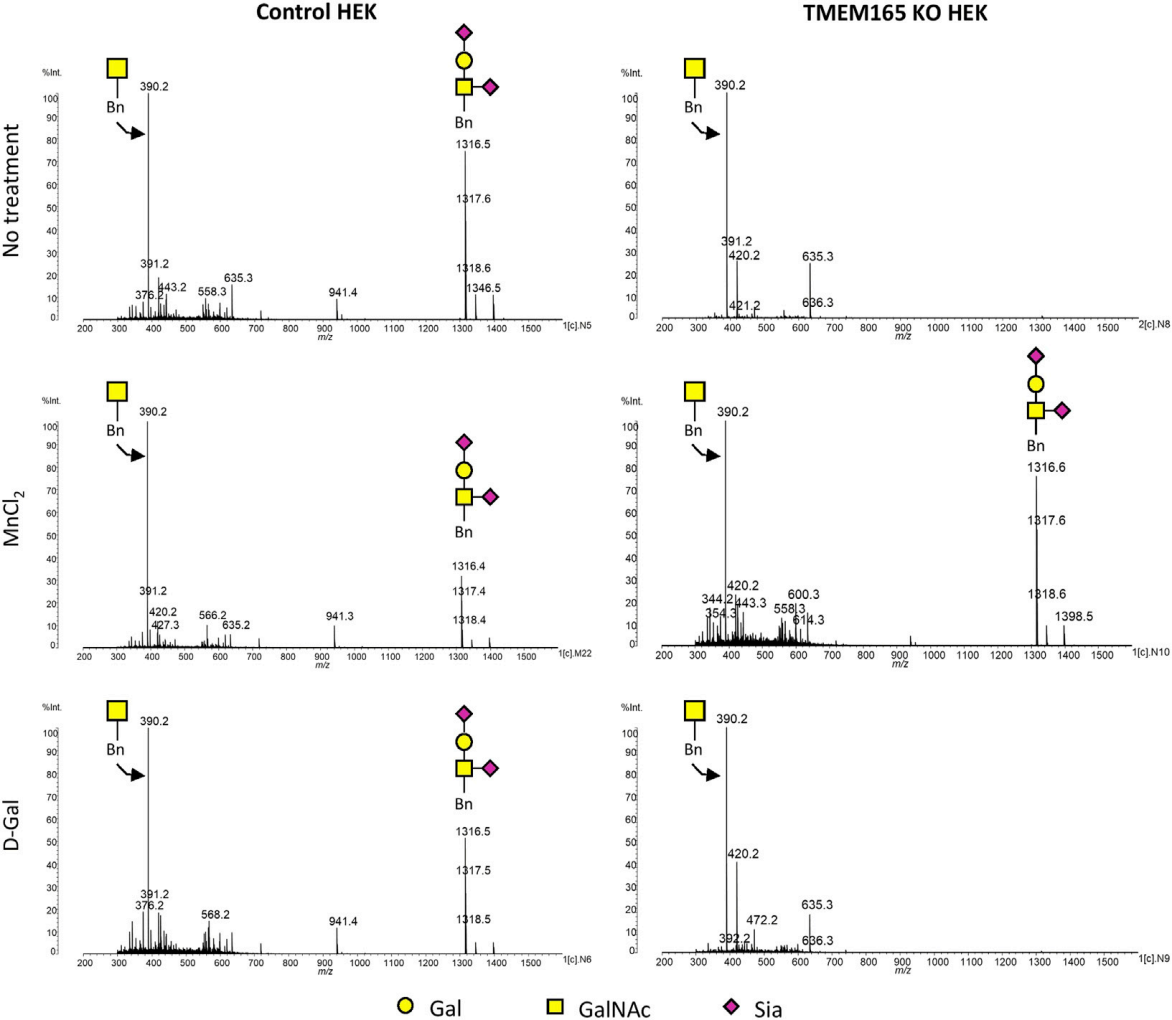
Mass spectrometry analysis of the Benzyl-GalNAc glycosylation status in HEK cells treated with MnCl_2_ or D-Galactose. MALDI-TOF MS spectra were obtained from control and TMEM165 KO HEK cells, treated or not with 1 mM D-Gal or 2.5 µM of MnCl_2_ and incubated with Bn-GalNAc at 250 µM for 3 days. Glycan structure of each peak of interest is schematized. Symbols used to represent the different sugar residues are indicated at the bottom of the figure. Gal, galactose; GalNAc, *N*-acetylgalactosamine and Sia, sialic acid.

As shown in Figure 4, MALDI-QUIT-TOF MS spectra from control and TMEM165 KO HEK cells treated or not with MnCl_2_ or D-Gal and incubated with Bn-GalNAc revealed the presence of two main species: unsubstituted Bn-GalNAc (m/z = 390) and Bn-GalNAc substituted with Gal and two sialic acid residues (m/z = 1316). In TMEM165 KO HEK cells, only unsubstituted Bn-GalNAc can be observed, thus confirming a strong *O*-galactosylation defect in those cells. Very interestingly, while MnCl_2_ supplementation was able to rescue the processing of *O*-glycan core 1 in TMEM165 KO HEK cells, as shown by the presence of fully sialylated Bn-GalNAc (m/z = 1316), D-Gal supplementation had absolutely no effect on Bn-GalNAc glycosylation status. Altogether these results confirm that the *O*-glycosylation defects detected in TMEM165 KO HEK cells can only be suppressed by supplementation with MnCl_2_ and not D-Gal.

### Strong GAG Defects in TMEM165 KO HEK Cells are Not Rescued Following D-Gal Supplementation

**FIGURE 5:**
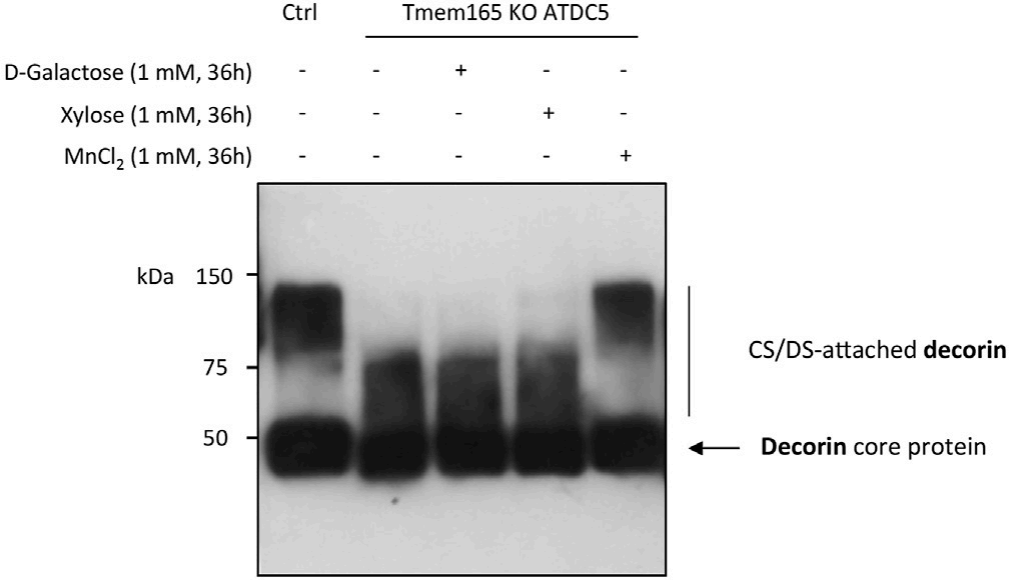
Manganese but not galactose or xylose supplementation rescues GAG elongation in Tmem165 KO ATDC5 cells. Wild-type (Ctrl) and TMEM165-knockout mouse ATDC5 cells were transfected with an expression vector coding for decorin and cultured in the presence or absence of D-galactose (1 mM), xylose (1 mM) or MnCl_2_ (1 µM). The electrophoretic migration profiles of decorin secreted in culture medium were then analyzed by western-blotting. The positions of decorin without (core protein) or with CS/DS chains are indicated on the right side of the western-blot.

Galactose is a key monosaccharide not only of the *N*-linked and *O-*linked mucin-type glycan structures but also of the core structure of GAGs (*O*-Xyl-Gal-Gal-GluA). In a recent paper, we have demonstrated a strong GAG defect in Tmem165 KO ATDC5 cells that was fully suppressed by 1 µM Mn^2+^ supplementation (Khan et al., 2021). To then investigate the impact of D-Gal supplementation on GAG synthesis, the elongation status of chondroitin sulfate (CS) chains of decorin was assessed in Tmem165 KO ATDC5 cells. Cells were first transiently transfected with decorin expression vector and analyzed under different conditions (either 1 mM D-Gal, 1 mM xylose or 1 µM MnCl_2_ for 36 h). As shown in Figure 5, the molecular weight of decorin produced in TMEM165 KO ATDC5 cells was significantly lower compared to control cells and did not change either with D-Gal or xylose supplementation. On the contrary, and as previously published (Khan et al., 2021), MnCl_2_ supplementation fully rescued the observed CS elongation on decorin. This result thus demonstrates that supplementation of Tmem165 ATDC5 cells with D-Gal does not suppress the observed GAG defect.

### Unraveling the Specificity of the D-Gal-Induced Golgi *N*-Linked Glycosylation Rescue in TMEM165 KO Cells

**FIGURE 6:**
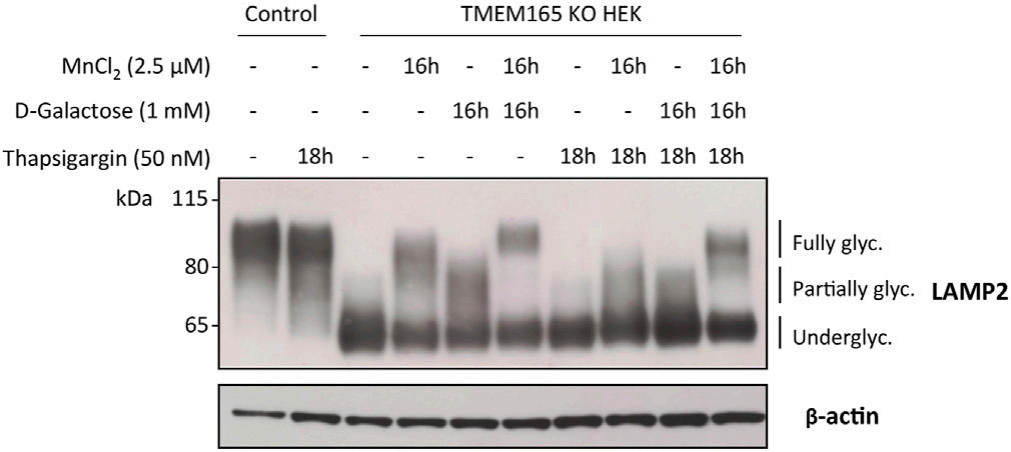
Impact of thapsigargin on LAMP2 and TGN46 glycosylation profiles in HEK cells treated with MnCl_2_ and/or D-Galactose. Western-blot of LAMP2 in control and TMEM165 KO HEK cells, treated for the indicated times with or without 2.5 μM MnCl_2_ and/or 1 mM D-Gal, in the presence or absence of 50 nM thapsigargin, a SERCA pump inhibitor. The electrophoretic migration positions of the fully- and under-glycosylated forms of proteins are indicated on the right side of the western blot (*n* = 3).

Our results here demonstrate that the D-Gal-induced Golgi glycosylation rescue in TMEM165 KO cells is restricted to the *N*-glycosylation pathway. We also previously demonstrated that the Mn^2+^-induced glycosylation rescue in TMEM165 KO HEK cells can be prevented by treatments with either thapsigargin or cyclopiazonic acid, both known to inhibit SERCA pumps in the endoplasmic reticulum (ER) (Houdou et al., 2019). However, the effect of such treatments on the D-Gal-induced *N*-linked glycosylation rescue in TMEM165 KO HEK cells was not evaluated. To tackle this point, TMEM165 KO HEK cells were pre-treated with either thapsigargin (50 nM) (Figure 6) or cyclopiazonic acid (100 μM) (Supplementary Figure S2) for 2 h and then incubated together with 1 mM D-Gal and/or 2.5 μM MnCl_2_ for 16 h. The electrophoretic migration profiles of LAMP2 were then assessed (Figure 6). As shown in Figure 6 and in Supplementary Figure S2, LAMP2 glycosylation was partially rescued following MnCl_2_ or D-Gal supplementation and completely annihilated when TMEM165 KO HEK cells were treated with thapsigargin or cyclopiazonic acid. Remarkably, thapsigargin and cyclopiazonic acid treatments had no effects when MnCl_2_ and D-Gal supplementations were combined, as observed by the presence of fully glycosylated LAMP2 forms. These results indicate that the combination of both compounds can overcome SERCA inhibition and that in absence of Mn^2+^, D-Gal supplementation alone has a rather poor effect on the Golgi *N*-linked glycosylation rescue in TMEM165 KO cells.

## Discussion

CDG are a group of inherited metabolic diseases where the glycosylation process is severely impaired. The extreme diversity of their biochemical defects often complicates any therapy. However, in some cases and for specific CDG-related defects, relatively simple treatments are possible, consisting of the oral intake of monosaccharides. To cite some examples, intakes of (i) D-Gal was successful in the treatment of PGM1-CDG and SLC35A2-CDG; (ii), L-fucose in the treatment of SLC35C1-CDG and (iii) D-mannose for MPI-CDG (Brasil et al., 2018; Sosicka et al., 2020; Verheijen et al., 2020). In the past few years, D-Gal supplementation particularly caught attention since abnormal galactosylation of *N*- and *O*-glycan structures, as well as glycolipids, was often found in CDG patients (Jaeken and Péanne, 2017). This resulting galactosylation defect may originate either from a decrease in the quantity of UDP-Gal (for PGM1-CDG), a defect in the import of UDP-Gal into the Golgi apparatus (for SLC35A2-CDG), or a defective Gal transfer onto the glycan structures by glycosyltransferases (for SLC39A8-CDG and TMEM165-CDG).

In both SLC39A8-CDG and TMEM165-CDG, glycosylation abnormalities interestingly originate from a primary defect of Mn^2+^ metabolism which can be alleviated by restoring physiological Mn^2+^ levels in body fluids and intracellular compartments thanks to MnCl_2_/MnSO_4_-H_2_O supplementations (Potelle et al., 2016; Park et al., 2018). Mn^2+^ is indeed a crucial cofactor of glycosyltransferases, most especially galactosyltransferases. In comparison to monosaccharide treatment, MnCl_2_ supplementation needs to be tightly controlled, as Mn^2+^ is toxic and Mn^2+^ overload or chronic exposure can result in the development of major neurodegenerative diseases (Parkinson’s like-syndrome, Huntington’s disease *etc.*) (O’Neal and Zheng, 2015; Erikson and Aschner, 2019; Harischandra et al., 2019)*.* Nevertheless, such Mn^2+^ supplementation (MnSO_4_-H_2_O) was successfully used with two patients suffering from SLC39A8-CDG with very low to undetectable Mn^2+^ blood levels (Park et al., 2018). In the published clinical trial, therapeutic MnSO_4_-H_2_O doses of 200 mg (20 mg/kg) and 600 mg (15 mg/kg) were established to be beneficial to both patients. Indeed, whereas no symptom of Mn^2+^-induced toxicity was detected in the patients, as checked with cranial MRI examinations, the MnSO_4_-H_2_O supplementation led to major clinical improvements with the suppression of many biochemical abnormalities (Park et al., 2018).

Although *in vitro* studies demonstrated that Mn^2+^ supplementation completely rescues the glycosylation of TMEM165-deficient cells (Potelle et al., 2016), such therapy is however difficult to apply to TMEM165-CDG patients whose Mn^2+^ blood levels were found normal. Interestingly, whereas both SLC39A8 and TMEM165-CDG present strong galactosylation defects of their *N*-linked glycans (Park et al., 2015; Morelle et al., 2017), we demonstrated that other Golgi glycosylation processes are affected in TMEM165-CDG patients, then contributing to the singularity of the TMEM165 deficiency (Morelle et al., 2017). In both SLC39A8 and TMEM165-CDG, the lack of galactosylation on patients’ serum *N*-glycoproteins was corrected by oral administration of D-Gal (between 0.5 and 1.5 g/kg per day) (Morelle et al., 2017; Sosicka et al., 2020; Verheijen et al., 2020).

In this study, we analyzed the differential effects of D-Gal supplementation on Golgi glycosylation defects caused by TMEM165 deficiency. Although D-Gal supplementation was quite efficient in suppressing the *N*-glycosylation defects, we demonstrate a poor efficiency on *O*-glycans and a total absence of effects on GAGs, as depicted by the electrophoretic mobility of decorin (Figure 5). This is also perfectly in line with our previous results on glycolipids where no effects of D-Gal supplementation were observed in TMEM165 KO cells (Morelle et al., 2017). This finding is very important in determining the management of TMEM165-CDG, as only clinical phenotypes associated with *N*-glycosylation defects could be rescued by D-Gal supplementation. The scope of such treatment is therefore limited and unsuitable to fully suppress the bone abnormalities due to GAG impairments in TMEM165-CDG patients (Bammens et al., 2015). Our results however demonstrate the complete suppression of the observed *N*-, *O*- and GAG defects by Mn^2+^ supplementation. Interestingly, we demonstrated the involvement of cyclopiazonic acid and thapsigargin (Tg)-sensitive pumps, likely ER SERCA pumps, in the rescue of TMEM165-associated glycosylation defects by Mn^2+^ (Houdou et al., 2019). Since the glycosylation defect in TMEM165 deficiency originates from a disrupted Golgi Mn^2+^ homeostasis (Potelle et al., 2016), it may be assumed that a too low Mn^2+^ concentration results in a lower catalytic activity of the Mn^2+^-dependent UDP-sugar glycosyltransferases, thus leading to massive and general Golgi glycosylation defects. Hence, Mn^2+^ supplementation provides the cells with the missing/required metal ion and thus fully rescues the glycosylation processes in the Golgi. The observed Mn^2+^ effect on Golgi glycosylation also demonstrates that the level of nucleotide-sugars, and particularly that of UDP-Gal, is not limiting. As such, any therapeutic strategy using MnSO_4_ seems to be the best option for TMEM165-CDG patients as it may alleviate the clinical phenotypes resulting from the overall glycosylation defects.

**FIGURE 7:**
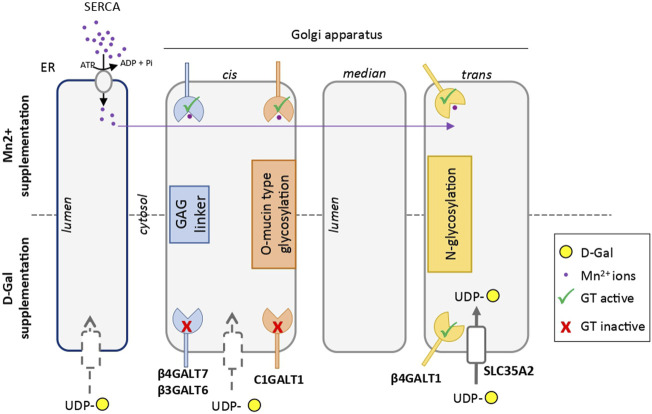
Scheme comparing the hypothetical mechanisms involved in the rescue of glycosylation induced by Mn^2+^ and D-galactose in TMEM165 KO cells. Endoplasmic *reticulum* (ER) and Golgi apparatus *cisternae* are represented. Galactosyltransferases involved in GAG linker synthesis, mucin-type *O*-glycosylation and *N-*glycosylation are indicated in blue, red and yellow, respectively. The UDP-Gal transporter SLC35A2 in the vicinity of the catalytic site of β4GALT1, thus explaining the selectivity of D-Gal for *N*-linked glycosylation, is represented with a white box in the *trans*-Golgi. The other putative UDP-Gal transporters in the ER and *cis*-Golgi apparatus are indicated by grey-dotted boxes. The Mn^2+^ ion is in purple. In absence of TMEM165 and after Mn^2+^ supplementation, cytosolic Mn^2+^ is pumped by SERCA in the ER lumen and redistributed within the Golgi to sustain, as a cofactor, the different galactosyltransferase activities.

From a fundamental point of view, the molecular mechanism involved in the specific rescue of the *N-*glycan structures by D-Gal supplementation is not well understood. A constant consequence following exogenous intake of Gal is the increase of the UDP-Gal cytoplasmic pool. This was observed in TMEM165 KO HEK cells (data not shown), as well as in PGM1-CDG. In this latter case, where the conversion of glucose-6-phosphate to glucose-1-phosphate is altered, the exogenous supply of D-Gal would promote the production of both UDP-Gal and UDP-glucose by action of the UDP-Gal 4-epimerase (GALE) (Varki et al., 2015). Interestingly, it was formerly reported that the beta 1,4 galactosyltransferase (B4GalT1) involved in the biosynthesis of *N*-glycan structures possesses two ion-binding sites: one for Mn^2+^ and one for Ca^2+^, both being used as cofactors (Powell and Brew, 1976). The authors also reported that the Mn^2+^/Ca^2+^ galactosyltransferase presents a 25-fold decreased affinity for UDP-Gal compared to Mn^2+^/Mn^2+^ enzyme. Since this feature was not reported for any other glycosyltransferase, it may be hypothesized that increasing the UDP-Gal pool by Gal supplementation would specifically impact the B4GalT1-mediated galactosylation process, and not the other Golgi glycosylation processes. Another explanation relies on the proximity of the UDP-Gal transporter SLC35A2 with the B4GalT1, as it was reported that SLC35A2 could form heteromeric complexes with B4GalT1 (Wiertelak et al., 2020). Following D-Gal supplementation, such interaction would result in an increased UDP-Gal concentration in the vicinity of the B4GalT1, thus explaining the selectivity of D-Gal for *N*-linked glycosylation (Figure 7).

Interestingly, our results support the involvement of thapsigargin- and cyclopiazonid acid-sensitive pumps in the *N*-glycosylation rescue resulting from D-Gal supplementation. Contrary to Mn^2+^, D-Gal supplementation alone was not sufficient for rescuing *N*-glycosylation in TMEM165 KO HEK cells treated with thapsigargin and cyclopiazonic acid, two SERCA inhibitors that prevent cytosolic Ca^2+^ and Mn^2+^ pumping in the ER (Vandecaetsbeek et al., 2011). This may be interpreted in two different ways: either a Gal supplementation has no effect when Mn^2+^ concentration within the secretory pathway is low, or such supplementation requires a proper ER/Golgi Ca^2+^ homeostasis to ensure optimal B4GalT1 activity. In support to the second possibility, it is worth noting that following SERCA inhibition by thapsigargin and cyclopiazonic acid, the Ca^2+^ gradient in organelles is disrupted. Based on the dependence of B4GalT1 on Ca^2+^ for its activity (Powell and Brew, 1976), improper/slower galactosylation reactions should then occur.

At last, the synergic effect of MnCl_2_ and D-Gal in rescuing glycosylation of TMEM165 KO HEK cells pre-treated or not with SERCA inhibitors is still not understood. Actually, the combined incubation of MnCl_2_ and D-Gal completely bypasses the effect of thapsigargin and cyclopiazonid acid. One can hypothesize that the increased UDP-Gal cytoplasmic pool due to the exogenous intake of D-Gal is able to chelate Mn^2+^ in the cytosol. Formation of such complexes would then facilitate the entry of Mn^2+^ into the Golgi and favor the catalytic activity of B4GalT1.

In conclusion, this paper demonstrates the differential impact of D-Gal or MnCl_2_ supplementations on Golgi glycosylation defects in the case of TMEM165 deficiency. Our results unambiguously demonstrate that MnCl_2_ supplementation can fully rescue the observed *N*- and *O*-linked and GAG glycosylations in TMEM165-deficient cells, while D-Gal supplementation can only partially rescue the *N*-linked glycosylation (Figure 7). Such results are particularly important for the establishment of guidelines for TMEM165-CDG therapies. Our results also suggest that the use of D-Gal in combination with MnCl_2_ potentiates MnCl_2_ effects. As such, the combination of D-Gal and MnCl_2_ would potentially be interesting in lowering the administered Mn doses and hence its toxicity.

## Data Availability

The original contributions presented in the study are included in the article/Supplementary Material, further inquiries can be directed to the corresponding author.

## Supplementary Material

Supplementary Figure S1
**(A)** VVL-fluorescein staining in control and COSMC KO HEK cells. Cells were fixed and labeled with VVL-fluorescein (green) before confocal microscopy visualization. DAPI staining (blue) was performed, showing nuclei. **(B)** Influence of MnCl_2_ and/or D-Gal supplementations on PNA-Cy5 staining in control and TMEM165 KO HEK cells. Cells were submitted to the indicated treatments, fixed and subjected or not to neuraminidase. Cells were stained with PNA-Cy5 (red) before confocal microscopy visualization. For clarification DAPI staining has been omitted (*n* = 3; p value<0,005).

Supplementary Figure S2 Impact of cyclopiazonic acid on the LAMP2 and TGN46 glycosylation profiles in HEK cells treated with MnCl2 and/or D-Galactose. Western-blot of LAMP2 in control and TMEM165 KO HEK cells, treated for the indicated times with or without 2.5 μM MnCl_2_ and/or 1 mM D-Gal, in the presence or absence of 100 μM cyclopiazonic acid, a SERCA pump inhibitor. The electrophoretic migration positions of the fully- and under-glycosylated forms of proteins are indicated on the right side of the western blot (*n* = 3).
