# Supplementary figures and images for: Differential Effects of D-Galactose Supplementation on Golgi Glycosylation Defects in TMEM165 Deficiency
Source: Front Cell Dev Biol. 2022 May 26;10:903953. doi: 10.3389/fcell.2022.903953 (PMC9178294; doi:10.3389/fcell.2022.903953)

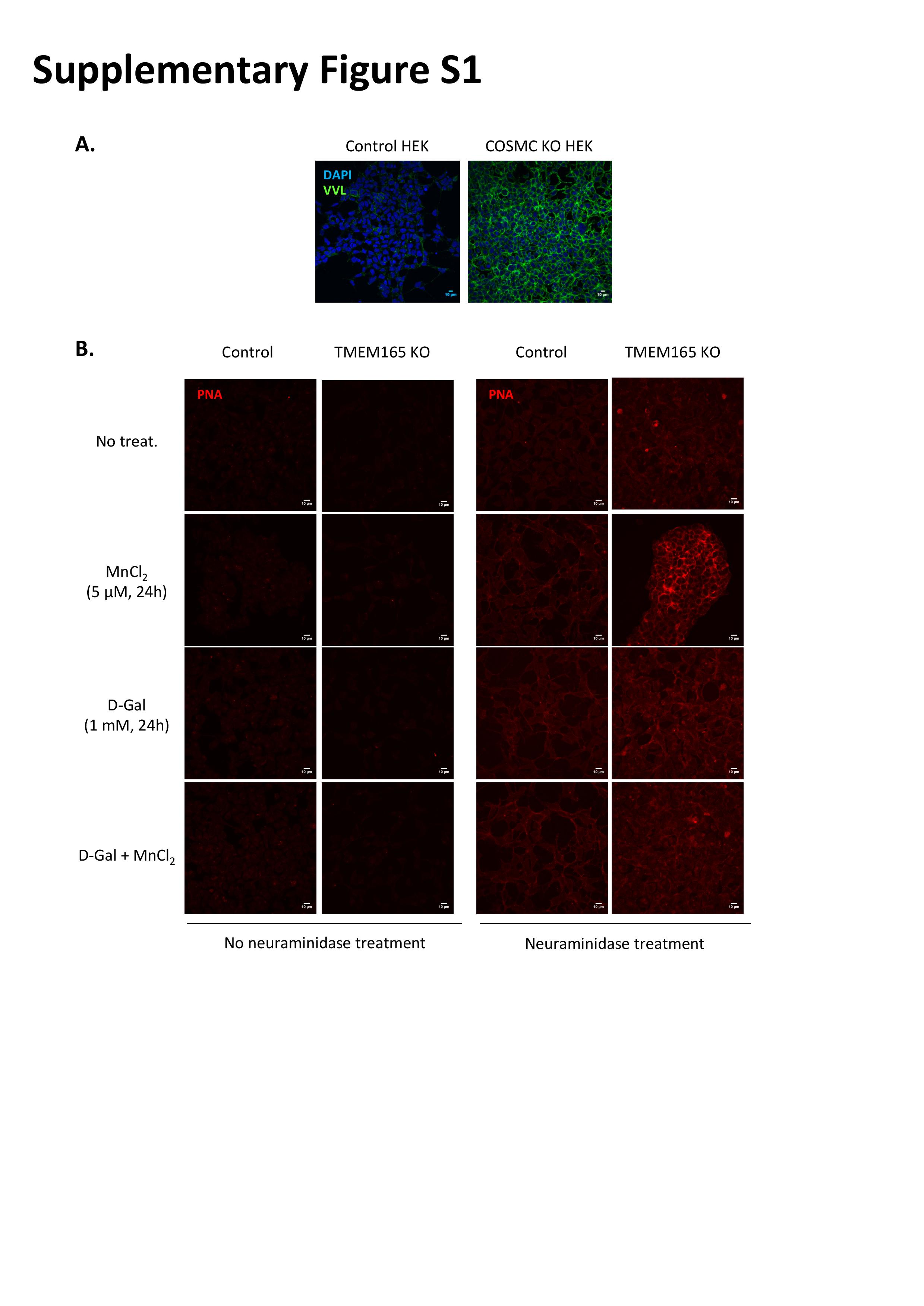

Supplement: Supplementary file 1 [file Image1.JPEG]

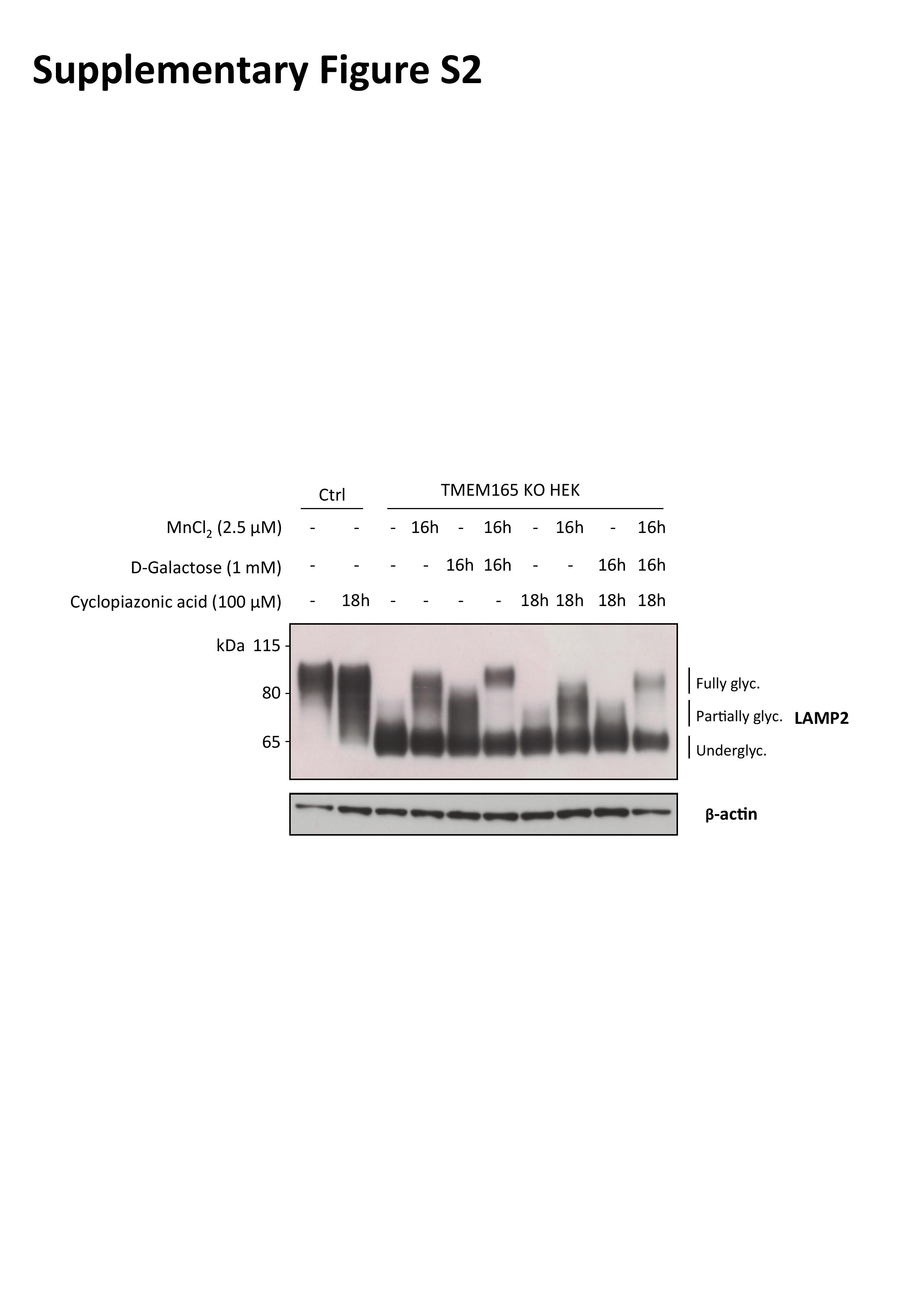

Supplement: Supplementary file 2 [file Image2.JPEG]
